# Learning-Induced Gene Expression in the Hippocampus Reveals a Role of Neuron -Astrocyte Metabolic Coupling in Long Term Memory

**DOI:** 10.1371/journal.pone.0141568

**Published:** 2015-10-29

**Authors:** Monika Tadi, Igor Allaman, Sylvain Lengacher, Gabriele Grenningloh, Pierre J. Magistretti

**Affiliations:** 1 Brain Mind Institute, Ecole Polytechnique Fédérale de Lausanne (EPFL), Lausanne, Switzerland; 2 Division of Biological and Environmental Sciences and Engineering, King Abdullah University of Science and Technology (KAUST), Thuwal, KSA, Saudi Arabia; Glasgow University, UNITED KINGDOM

## Abstract

We examined the expression of genes related to brain energy metabolism and particularly those encoding glia (astrocyte)-specific functions in the dorsal hippocampus subsequent to learning. Context-dependent avoidance behavior was tested in mice using the step-through Inhibitory Avoidance (IA) paradigm. Animals were sacrificed 3, 9, 24, or 72 hours after training or 3 hours after retention testing. The quantitative determination of mRNA levels revealed learning-induced changes in the expression of genes thought to be involved in astrocyte-neuron metabolic coupling in a time dependent manner. Twenty four hours following IA training, an enhanced gene expression was seen, particularly for genes encoding monocarboxylate transporters 1 and 4 (MCT1, MCT4), alpha2 subunit of the Na/K-ATPase and glucose transporter type 1. To assess the functional role for one of these genes in learning, we studied MCT1 deficient mice and found that they exhibit impaired memory in the inhibitory avoidance task. Together, these observations indicate that neuron-glia metabolic coupling undergoes metabolic adaptations following learning as indicated by the change in expression of key metabolic genes.

## Introduction

Memories can be broadly classified into short term memory (STM) and long term memory (LTM). While STM involves post-translational modification of pre-existing molecules to modify the efficiency of synaptic transmission, LTM formation is associated with changes in gene expression and *de novo* protein synthesis [1–3]. Learning initiates a cascade of events in the brain that requires gene activation, post-translational modifications and downstream alterations in neurons to store the newly acquired information [4].

A ubiquitous feature of neuronal activity is that it requires energy. The coupling between neuronal activity and energy metabolism is at the basis of functional brain imaging techniques, which detect increases in energy demands associated with synaptic signaling. At the metabolic level, the astrocyte–neuron lactate shuttle (ANLS) model summarizes a whole chain of events involved in the coupling of neuronal and metabolic activity upon functional brain activation [5–8]. During increased synaptic activity, synaptically-released glutamate is avidly taken up via a Na^+^-dependent mechanism by specific glial glutamate transporters. The resulting increase in [Na^+^]_i_ activates the Na/K ATPase (particularly by mobilizing its alpha2 subunit), thereby increasing ATP consumption [9], glucose uptake and glycolysis in astrocytes. This in turn leads to a large increase in the production of lactate which is released into the extracellular space and could be used as an energy substrate by neurons for oxidative-derived ATP production [5]. Another important astrocyte-neuron metabolic interaction occurs via glycogen metabolism. In the mature central nervous system, glycogen storage occurs exclusively in astrocytes [10–12] and astrocytic glycogen can be subsequently broken down (glycogenolysis) by the astrocyte-specific enzyme, glycogen phosphorylase [11,12]. Previous studies have shown the importance of glycogenolysis and the subsequent release of lactate from astrocytes in memory formation and long-term potentiation [13,14].

The underlying hypothesis that we were interested to test, was whether the plastic changes that have been revealed for the synaptic machinery following learning are accompanied by corresponding changes in the expression of genes involved in energy metabolism, in particular the neuron-glia metabolic coupling. First, we examined regional brain metabolic activity in several brain structures following context dependent inhibitory avoidance (IA) learning. In a second step, we analyzed expression of genes that encode proteins involved in energy metabolism at several time-points following IA task in the hippocampus, one of the regions that exhibited increased activity upon metabolic mapping. To obtain a more direct evidence for a functional role of genes associated with neuron-glia metabolic coupling in learning and memory, we studied the cognitive behavior of mice deficient for the monocarboxylate transporter 1 (MCT1), a transporter known to transport lactate across the plasma membrane of astrocytes [8,15].

## Materials and Methods

### Animals

All animal experiments of this study were approved by the ‘Commission pour les expériences sur animaux de l’état de Vaud’ [Ethics committee for animal experiments of the canton Vaud], Switzerland and the authorizations (no. VD2044.1 and VD2044.2) were issued by the ‘Service de la consommation et des affaires vétérinaires’ [Cantonal veterinary office]. All animal experiments performed in Switzerland underly the 'Loi fédérale sur la protection des animaux (LPA)’ [Federal law on the protection of animals] (455) and the ‘Ordonnance sur la protection des animaux (OPAn)’ [Ordinance on the protection of animals] (455.1) and the ‘Ordonnance sur l'expérimentation animale’ [Ordinance on animal experimentation] (455.163).

Eight to twelve weeks old C57BL/6 male mice (Charles River Laboratories, L'Arbresle, France) and heterozygous Slc16a1 knock-in/LacZ male mice [B6.129OlaHsd-MCT1tm(lacZ)Syle], backcrossed for at least 6 generations with C57BL/6 mice (referred as MCT1^+/-^ mice [16,17]), were group-housed in IVC type cages under standard housing conditions (22°C ±1, 12:12-h light: dark cycle). Pellet food and water were available ad libitum. Prior to experimentation mice were habituated for one week to these conditions. On the day of the experiment, the animals were allowed to habituate to the experimental room for half an hour before beginning the behavioral test. All animals were monitored at least two to three times each week to assess well-being, normal weight (according to the body growth curve for C57BL/6, The Jackson Laboratory) and to monitor signs of distress.

### Step through inhibitory avoidance

Mice were trained to learn context dependent avoidance behavior using a single trial IA test (littermate comparison for MCT1 deficient mice). An automated shuttle box MED PC-IV (Med Associates, St. Albans, USA) consisting of two identical chambers in sound-attenuating boxes (425 x 635 x 600 mm) separated by a guillotine door was used for the test. The floor of each chamber was made of stainless-steel grid and connected to a scrambled electric foot shock generator. To condition the animals to fear, a conditioned stimulus (CS; the context) was paired with an unconditioned stimulus (US; i.e. foot shock). After acclimatization for a minute in the light compartment, the guillotine door was automatically opened to start the acquisition trial. Once the animal was in the dark compartment with all four paws, the door closed automatically and a foot shock was delivered (0.5mA) for a duration of 2 seconds. Transfer latency was recorded by infrared sensors that monitor the transfer of the animal from light to the dark compartment (from the time the door was lifted). As the computerized door was closed upon transfer; the mouse was subjected to the full duration of the electric shock. Ten seconds after administering the foot shock, the mouse was removed from the chamber and returned to its home cage. Twenty four hours after the training trial, half of the mice (n = at least 7/group for each experiment) were subjected to retention testing. Here, the trained mouse was then again placed in the bright chamber and the latency to re-enter the dark compartment was recorded as the transfer latency for the retention trial. The cut-off time for retention testing was set to 300 seconds. The learning ability of the animal was determined by an increase in the transfer latency during the retention testing. Mice were divided into 2 groups: 1) CS-US group, mice were trained as described above; 2) CS group, mice received a training session in the absence of a foot shock. A shock only (US) group was also included in one series of experiments to identify the effects of shock/stress on gene expression. Mice in the US group directly received a foot shock in the dark compartment and were immediately removed from the box after the foot shock.

### [^14^C]-2-deoxyglucose technique

One day after IA training, mice received an intraperitoneal injection of [^14^C] 2-deoxyglucose [([^14^C] 2DG), Hartmann Analytic, Braunschweig Germany)] [18] and were returned to their home cage for 15 min followed by IA retention test, lasting for approximately 5 min. After the test, the animals were returned to their home cage for 10 min and then sacrificed by lethal IP injection of pentobarbital (150 mg/kg) (a total time of around 30–35 min after the [^14^C] 2DG injection). The brains were then rapidly extracted and frozen in dry ice [18]. Coronal brain sections of 20 μm were obtained using a cryostat and processed for autoradiography together with ^14^C standard microscales (Amersham Biosciences Europe GmbH, Freiburg, Germany). After exposure, the films were developed and the brain sections stained with cresyl violet. Images of the brain slices were acquired and autoradiograms along with the corresponding stained images were digitized with an image densitometry system (MCID, Imaging Research, St. Catharines, Canada). Using the MCID software, the average optical density (OD) of the hippocampus, amygdala [i.e. basolateral amygdala (BLA) and the lateral nucleus (LA)], anterior cingulate cortex (ACC), prelimbic (PL) and infralimbic (IL) cortex was determined. Regions were based on the mouse brain atlas from Hof et al. [19]. Optical density measurements from the selected regions of interest (ROIs) were obtained from all consecutive sections on which a given brain structure could be identified. For each ROI, a mean OD value of all consecutive sections was calculated for each mouse. The OD values for the white matter (corpus callosum) was measured on each examined section and used as a background intensity reference to normalize data between animals.

### Quantitative-polymerase chain reaction (Q-PCR)

C57BL/6 mice were sacrificed either at 3, 9, 24, 72 hours post training or 3 hours post retention testing depending on the experimental group they belonged to. The brains were removed, frozen in dry ice and coronally sectioned (300 μm) using a cryostat. Dorsal hippocampus and somatosensory cortex were dissected on a refrigerated micro plate [19] and total RNA was isolated using the NucleoSpin RNA II kit (Macherey-Nagel, Oesingen, Switzerland) as per the manufacturer's instructions. RNA concentration was determined using NanoDrop ND-1000 spectrophotometer (Thermo Fischer scientific, Reinach, Switzerland). The first strand of cDNA was synthesized from total RNA (60 min at 37°C, followed by 5 min at 95°C) using the High Capacity RNA-to-cDNA Reverse Transcription system (Applied Biosystems, Foster City, USA). Resulting cDNA was amplified by quantitative PCR (qPCR) with the ABI PRISM 7900 system (Applied Biosystems). PCR mix was composed of 0.3 ng of cDNA and 200 nM forward and reverse primers in 10 μl of 1× SYBR Green PCR Master Mix (Applied Biosystems). Primer sequences were designed using a primer designing tool (http://www.ncbi.nlm.nih.gov/tools/primer-blast/) or primer Express 3.0 software (Applied Biosystems) and oligonucleotides were synthesized by Microsynth (Balgach, Switzerland). Forward and reverse primer sequences for the genes used for the study were as follows: *Beta-actin* (β-actin) (NM_007393): 5’-GCTTCTTTGCAGCTCCTTCGT-3’ and 5’-ATATCGTCATCCATGGCGAAC-3’; *Lactate dehydrogenase A (Ldha)* (NM_010699): 5’- TTGTCTCCAGCAAAGACTACTGTGT-3’ and 5’-TTTCGCTGGACCAGGTTGAG-3’; *Lactate dehydrogenase B (Ldhb)* (NM_008492): 5’-GCAGCACGGGAGCTTGTT-3’ and 5’-CAATCTTAGAGTTGGCTGTCACAGA-3’; *Monocarboxylic acid transporter type 1 (MCT1*, *Slc16a1)* (NM_009196): 5’-AATGCTGCCCTGTCCTCCTA-3’ and 5’-CCCAGTACGTGTATTTGTAGTCTCCAT-3’; *Monocarboxylic acid transporter type 2 (MCT2*, *Slc16a7)* (NM_011391): 5’-CAGCAACAGCGTGATAGAGCTT-3’ and 5’-TGGTTGCAGGTTGAATGCTAAT-3’; *Monocarboxylic acid transporter type 4 (MCT4*, *Slc16a3)* (NM_030696): 5’-TCTGCAGAAGCATTATCCAGATCTA-3’ and 5’- ATGATGAGGGAAGGCTGGAA-3’; *Facilited glucose transporter type 1 (Glut1*, *Slc2a1)* (NM_011400): 5’-CCAGCTGGGAATCGTCGTT-3’ and 5’- CTGCATTGCCCATGATGGA-3’; *Facilitated glucose transporter type 3 (Glut3*, *Slc2a3)* (NM_011401): 5’-GAGGAGAACCCTGCATATGATAGG-3’ and 5’-CAAAGCTCATGGCTTCATAGTCA-3’; *ATPase*, *Na* ^*+*^ */K* ^*+*^ *transporting*, *alpha 2 polypeptide (Na/K alpha2*, *ATP1A2)* (NM_178405): 5’-GAGACGCGCAATATCTGTTTCTT-3’ and 5’-ACCTGTGGCAATCACAATGC-3’; *Glucan (1*,*4-alpha-) branching enzyme 1 (Gbe1)* (NM_028803): 5’-GCTGAGGCTTTTGAACATAATGG-3’ and 5’-TGCAGATCCACATTCTGAAGGA-3’; *Glycogen synthase type 1*, *muscle (Gys1)* (NM_030678): 5’-TGCAGCAGCTCACTGTGCCC-3’ and 5’-AGTTGAGCCGGGCCAATGCC-3’; *Glycogen synthase type 2*, *liver (Gys2)* (NM_145572): 5’-CCTTGTCGGTGACATCCCTTGGT-3’ and 5’-TGTCGTTTTGGCCTTGGTCTGGAT-3’; *UDP-glucose pyrophosphorylase type 2 (Ugp2)* (NM_139297): 5’-ACCCAATGGGAAGCGCTGTGA-3’ and 5’-TGTGCTTTGGGCACTTGAGCG-3’; *Amylo-1*,*6-glucosidase*, *4-alpha-glucanotransferase (Agl)* (NM_001081326): 5’-TGAGTGGTCGGCTTATTTCAAG-3’ and 5’-GTGGGATCTGCTTCAGGTAGAAG-3’; *Phosphoglucomutase type 1 (Pgm1)* (NM_025700): 5’-TGGGTATGGACGCGCGACTG-3’ and 5’-GGCCCCAAAGCATTTCCGCA-3’; *Phosphoglucomutase type 2 (Pgm2)* (NM_028132): 5’-TTTGCACGCAGCATGCCCAC-3’ and 5’-CTTCCAGCCAGTTGGGGTCTCA-3’; *Glycogen phosphorylase*, *brain (Pygb)* (NM_153781): 5’-GCTGCTCAACTGCCTACACATT-3’ and 5’-AACAGTCCTGGGCACAAAGG-3’; *Protein phosphatase 1*, *regulatory (inhibitor) subunit 3C (PPP1r3C*, *PTG)* (NM_016854): 5’-TGCCTCTCGGTCCAATGAG-3’ and 5’-GGCATGACGGAACTTGTCAA-3’; *Glycogenin (Gyg)* (NM_013755): 5’-ACACCTTCACCACCAACGTCTT-3’ and 5’-GCTCCTGAGACATGTTCCATCAT-3’; *Phosphorylase kinase beta (Phkb)* (NM_199446): 5’-TGGGCCTTGGCGCTAGCATAC-3’ and 5’-GTGCTCCAGCTCATGGGTCCG-3’; *Pyruvate carboxylase (Pcx)* (NM_008797): 5’-TCCCGTTCCAGATGCTACTGA-3’ and 5’-ATTCTCTTTGGCCACCTCACA-3’; *Pyruvate dehydrogenase kinase*, *isoenzyme 1 (Pdk1)* (NM_172665): 5’-TTCACGTCACGCTGGGCGAG-3’ and 5’-GCACAGCACGGGACGTTTCA-3’; *Pyruvate dehydrogenase kinase*, *isoenzyme 2 (Pdk2)* (NM_133667): 5’-GTTCTCCCCGTCCCCGTTGT-3’ and 5’-GCTCCTGCCGGAGGAAAGTGA-3’; *Pyruvate dehydrogenase kinase*, *isoenzyme 3 (Pdk3)* (NM_145630): 5’-TCGAGCGCTACTCCCGCTTCT-3’ and 5’-CTCTCTCATGGTGTTAGCCAGTCGC-3’; *Pyruvate dehydrogenase kinase*, *isoenzyme 4 (Pdk4)* (NM_013743): 5’-TCTCGACCGCGCTCCTGACC-3’ and 5’-GGGCAGCTCTTGCCGCAGAA-3’; *Glucose-6-phosphate dehydrogenase* X-linked *(G6pdx*, *G6pdh)* (NM_008062): 5’-CCCAGCCCATCCCCTATG-3’ and 5’-CTCGTACTGGAAGCCCACTCTCT-3’; *Transaldolase 1 (Taldo1)* (NM_011528): 5’-GAAAGGAGCTGGAGGAACAG-3’ and 5’-CTGGGCGAAGGAGAAAAGC-3’; *Transketolase (Tkt)* (NM_009388): 5’-CACCTTCTCGGAGCTCTTCAA-3’ and 5’-AAAGTACTGCAGAAGGGCACTGT-3’. The specificity of PCR amplification for each set of primers was checked by the presence of a single sharp peak in the melting curve analysis. Data were computed using the sequence detector software SDS 2.3 (Applied Biosystems) and analyzed using a macro developed by the genomic platform of Geneva University (Frontiers in Genetics, UNIGE). Gene expression levels were normalized to β-actin values. Delta-C_T_ relative quantification (ΔΔC_t_) was used and data were expressed as percentage of control.

### Western blot analysis

C57BL/6 mice were sacrificed 24 hours post IA training or 6 hours post retention testing. The brain was removed, frozen in dry ice and coronally sectioned (300 μm) using a cryostat. Dorsal hippocampus was dissected on a refrigerated micro plate using the Hof mouse atlas as a reference [19]. Protein extracts were prepared by homogenization in extraction buffer (in mM: 30 HEPES, 210 sucrose, 40 NaCl, 2 EGTA, SDS 2%) containing protease (Roche, Rotkreuz, Switzerland) and phosphatase (Sigma-Aldrich, Buchs SG, Switzerland) inhibitor cocktails. The supernatant was quantified by BCA assay (Pierce Biotechnology, Lausanne, Switzerland). Ten μg of whole protein homogenates from each animal was subjected to SDS-PAGE, blotted and probed with protein-specific antibodies. Proteins were resolved on NuPAGE 15% Bis-Tris minigels using MES SDS running buffer (Invitrogen, Basel, Switzerland) and transferred to Immobilon-P PVDF membranes (Millipore, Zug, Switzerland). The membranes were blocked for 1 hour in PBS containing 0.1% Tween 20 (PBST) and 5% dry milk and then incubated overnight at 4°C with antibodies directed against MCT1 (anti-chicken polyclonal; AB1286 Millipore; 1/15’000 dilution), MCT4 (anti-rabbit polyclonal; H-90 Santa Cruz Biotechnology, Santa Cruz, USA; 1/5’000 dilution), Glut1 (anti-mouse monoclonal; ab652, Abcam, Cambridge, UK; 1/5’000 dilution), Na/K ATPase alpha 2 isoform (anti-rabbit polyclonal; NBP1-00937, Novus Biologicals, Cambridge, UK; 1/3’000 dilution) or Pygb (anti-mouse polyclonal; 12075-1-AP Proteintech, Cicago, USA; 1/2'000 dilution). After washing with PBST, the blots were probed with the appropriate HRP-conjugated secondary antibody (GE Healthcare, Glattburg, Switzerland) diluted 1:15’000 in PBST plus 5% dry milk. After extensive washing with PBST, the peroxidase activity was detected by chemiluminescence using the ECL detection system (GE Healthcare). The membranes were never stripped to avoid loss of protein and in the case of proteins with the same molecular weight, separate gels were loaded. β-actin or β-tubulin expression was assessed to ensure equal loading. β-tubulin was used where the tested proteins molecular weight overlapped with that of β-actin. Membranes were washed several times with PBST and probed as described above using a mouse monoclonal antibody against β-actin diluted 1:500’000 (Sigma-Aldrich) or β-tubulin diluted 1:40’000 (Sigma-Aldrich). Immunoreactivity was detected using the ChemiDoc XRS system (Bio-Rad, Cressier, Switzerland). Densitometry analysis on the bands was calculated using Quantity One 4.2.3 software (Bio-Rad). Each band was normalized to β-actin or β-tubulin as determined in the corresponding sample. Protein changes were represented as a percentage of the mean control value. Protein measurements were performed in the linear range for all immunoblot assays.

### Statistical analysis

All results are presented as the mean ± SEM and significance was accepted at p < 0.05 for all statistical tests. Data were analyzed for statistical significance by two tailed unpaired Student's t test, or by two-way ANOVA using the Prism 4.0; GraphPad Software. Statistically significant two-way ANOVAs were followed by a Bonferroni's multiple-comparison test.

## Results

**Fig 1:**
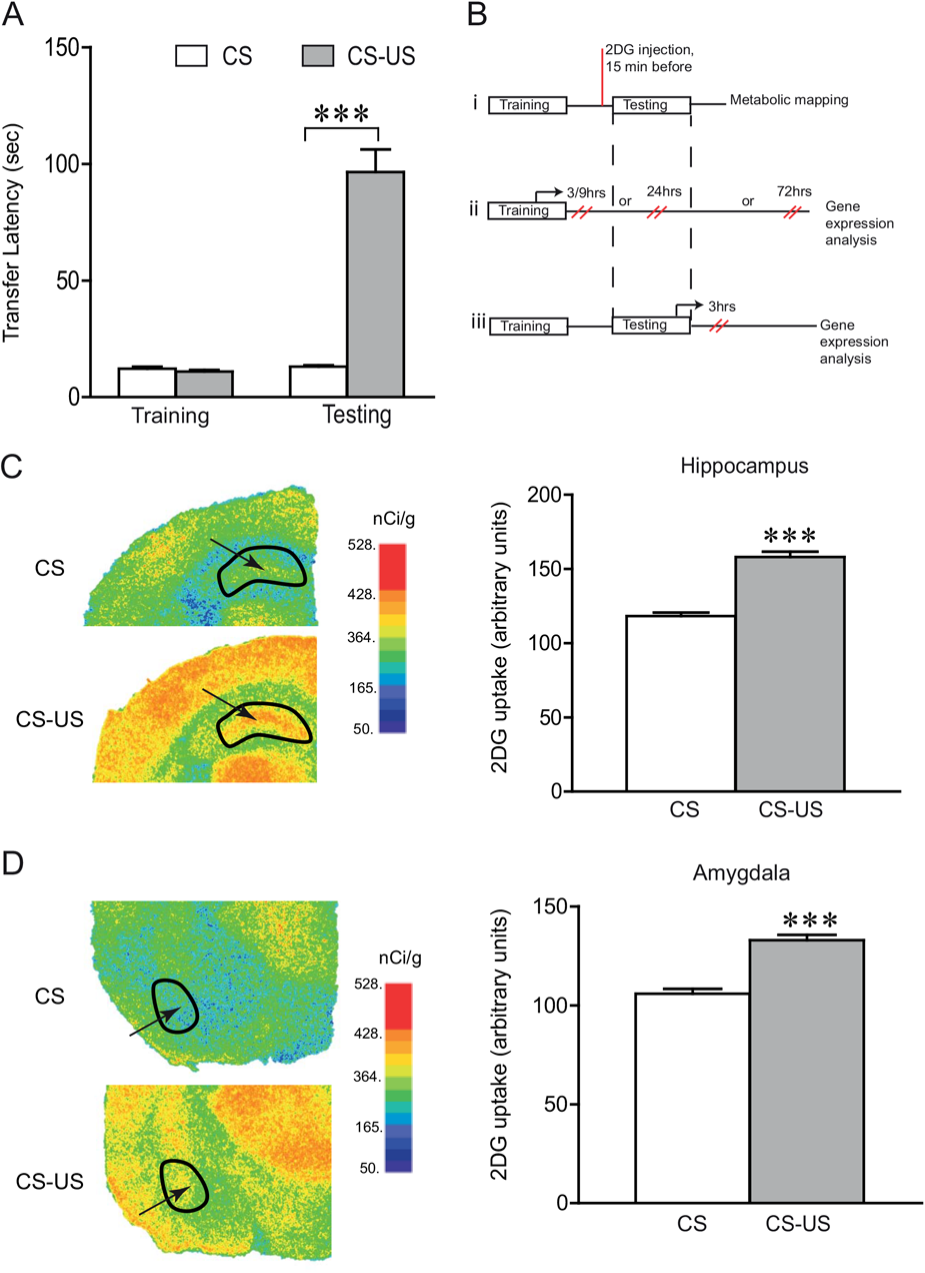
Metabolic mapping of the brain following inhibitory avoidance (IA) learning. **(A)** IA learning: Mice subjected to IA training (CS-US) show long-term memory while those exposed only to the training session without a foot shock (CS) do not acquire the task. Step-through transfer latencies during training and on the retention testing, 24 hours after training are shown. The baseline latencies were not significantly different between the two groups (P> 0.05). During the retention testing, the CS-US group had longer mean step-through latency than the control mice. Data were statistically analyzed with unpaired t-test (***P < 0.001 vs CS group, n = 40/group). **(B)** Experimental protocol: Schematic representation of the IA task and time structure of the different experimental groups used for the study. (i) [^14^C] 2DG technique allowed metabolic mapping of the brain regions activated during retention testing. (ii, iii) Gene expression analysis was performed either after inhibitory avoidance training or testing and accordingly animals were sacrificed either at 3, 9, 24 or 72 hours following training or 3 hours after retention testing. Quantitative mRNA levels for genes related to brain energy metabolism were probed in the dorsal hippocampus. **(C, D)** Functional activation of the brain during IA retention testing: Representative digitized autoradiograms of the distribution of [^14^C] 2DG uptake on brain sections from the CS and CS-US group. Arrows point to dorsal hippocampus (C) and basolateral amygdala (BLA, D). Outlines mark boundaries in which optical density was measured. In the autoradiographs the level of [^14^C] 2DG uptake is shown in a color scale ranging from blue (no uptake at all) to red (maximal uptake). [^14^C] 2DG uptake was quantified in the region of interests (ROIs) during retention testing. [^14^C] 2DG uptake (nCi/g) was evaluated by film densitometry and is expressed as mean ± SEM. CS-US group exhibited increased glucose utilization on the retention test, in the hippocampus (C, unpaired t-test with Welch’s correction, P< 0.0001, t = 9.445, df = 14) and in the amygdala (BLA and LA) (D, unpaired t-test with Welch’s correction P< 0.0001, t = 7.416, df = 14).

In this study, we investigated some aspects of the molecular mechanisms underlying learning and memory, by focusing on genes involved in energy metabolism, with a particular emphasis on neuron-glia metabolic coupling. We used IA task to study the expression of genes involved in brain energy metabolism following memory formation. This task requires hippocampal-dependent learning and transcription and has been widely used to characterize the biochemical requirements for memory formation, particularly in the hippocampus [20–22]. Two groups were used for this study. A conditioned group of mice (CS-US group) received a footshock after they entered into the dark compartment in the training session. The control (unconditioned) animals were treated similarly, except that they did not receive a footshock in the dark compartment (CS group). Twenty-four hours following training, the CS-US group exhibited 7 times longer latencies (96.5 ± 9.7 vs 13.1 ± 0.5 sec) to enter the dark compartment (Fig 1A), as compared to the CS group. This indicates that only the CS-US group has learned to associate the stepping through to the dark chamber with the aversive foot shock.

To identify the brain regions that were activated when IA long term memory is retrieved, we then performed brain metabolic mapping using radioactive [^14^C] 2DG autoradiographic technique [18]. Since the development of the [^14^C] 2DG autoradiography method in the 1970s [18], it has been extensively used to obtain functional maps of learning related specific brain areas such as in classical Pavlovian conditioning [23,24], in fear extinction learning [25] or in different working memory tasks [26,27]. Regions of interest (ROIs) were chosen for [^14^C] 2DG quantification based on previous studies showing regions involved in fear memory i.e., hippocampus, amygdala, anterior cingulate cortex (ACC), prelimbic (PL) and infralimbic (IL) cortex [28–32]. When tested for retention (here referred to the time between learning and testing of memory retrieval) of LTM, 24 hours following training (Fig 1B), the CS-US group showed 33 ± 3.5% increased glucose utilization in the hippocampus (Fig 1C), 25 ± 2.7% increase in the amygdala (Fig 1D), 14 ± 0.1% increase in the ACC and 11± 0.07% increase in the IL and PL cortex (unpaired t-test with Welch’s correction, P < 0.05, data not shown) compared to the CS group. The observed increases in metabolic activity in the hippocampus, amygdala, ACC, IL and PL cortex after IA learning identify these regions as being activated, and provide a mapping to guide the analysis of the learning- and activation-induced gene expression in the context of LTM formation.

In order to determine if such gene expression changes involve genes related to brain energy metabolism, we performed a detailed gene expression analysis in the hippocampus at several time points post IA training and retention testing (Fig 1B). Following IA retention testing, enhanced glucose utilization was observed in the hippocampus (Fig 1C). For gene expression analysis, we focused specifically on the dorsal as opposed to ventral region of the hippocampus because of the well characterized involvement of dorsal hippocampus' role in memory generally and in contextual fear in particular [33–35]. Studies based on more detailed time courses have shown that long-term memory formation is associated with an early and late phase of gene expression [36,37]. We therefore, analyzed the temporal dynamics of brain energy metabolism related gene expression over different time intervals following IA task. Dorsal hippocampal gene expression profiles in the CS and CS-US group were analyzed either at 3, 9, 24 or 72 hours after training or 3 hours after retention testing. Expression levels of genes involved in energy metabolism focusing on Astrocyte-Neuron Lactate Shuttle (ANLS) and glycogen metabolism were determined following IA learning.

### Genes involved in energy metabolism are modulated following IA learning

**Fig 2:**
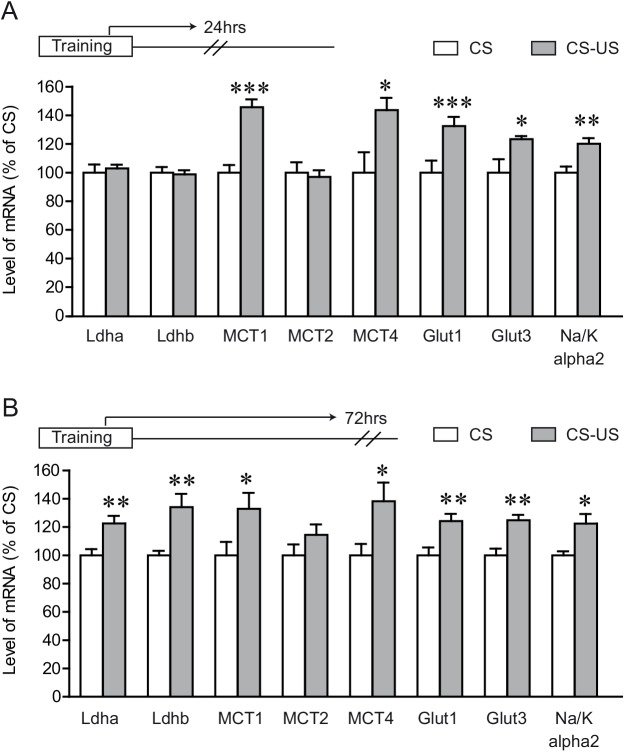
IA training results in enhanced and prolonged expression of ANLS related genes. Dorsal hippocampal tissue was collected 24 hours following inhibitory avoidance training **(A)** or 72 hours following inhibitory avoidance testing **(B)** and mRNA expression levels for the ANLS related genes were assessed by quantitative Q-PCR. Results are expressed as percentage of control values (CS group) and are means ± SEM. Data were statistically analyzed using two-tailed Student’s t test, * P < 0.05, ** P < 0.01, *** P < 0.001 vs CS group, n = 8/group for condition A and 7/group for condition B.

Transfer of energy substrates from astrocytes to neurons is the central point of the ANLS [5]. According to the ANLS, lactate produced preferentially by the astrocytes, is shuttled via specific monocarboxylate transporters (*MCTs*) from the astrocytes to the active neurons. Previous work has shown that lactate transport from astrocytes into neurons is necessary for long term memory formation [13] as well as for short term spatial working memory [14]. When examining ANLS related genes, no gene expression changes were seen 3 and 9 hours post IA training (data not shown). However, we found a late phase (24 hours following training) of enhanced dorsal hippocampal expression of several ANLS related genes in the CS-US group (Fig 2A, vs CS group, P < 0.05 for each unpaired t-test, n = 8). Specifically we observed a 46 ± 5.4% increase in mean mRNA levels of monocarboxylate transporter 1 (*MCT1*), 44 ± 8.3% increase in astrocytic monocarboxylate transporter 4 (*MCT4*), a 32 ± 6.4% increase in astrocytic glucose transporter 1 (*Glut1*), and a 20 ± 3.4% increase in Na/K-ATPase alpha2 subunit (*Na/K alpha2*). Note that a 23 ± 2.3% increase in neuronal glucose transporter 3 (*Glut3*) was also observed, while the neuronal *Na/K alpha3* subunit expression remained unaltered (data not shown). In contrast to the dorsal hippocampal gene expression results, no induction of ANLS related genes was observed in the somatosensory cortex of the CS-US group, 24 hours after IA training (S1 Table). Furthermore, to investigate if shock alone had any effect on the expression/modulation of genes related to brain energy metabolism following IA task, a US group (shock only) was included to the experimental paradigm. Mice in the US group were directly placed in the dark compartment where they received a foot shock and were immediately removed from the box after the foot shock to block context-shock association [38–40]. When compared to the CS group, there was no induction of ANLS related genes in the US group, 24 hours after IA training (S2 Table).

It has been shown that high levels of phosphorylated cAMP response element-binding protein (CREB) persist in the hippocampus for several hours following IA training [39,41]. We therefore extended our gene expression analysis 72 hours post IA training. We observed that the late phase of enhanced expression of ANLS related genes seen at 24 hours post training (Fig 2A) persisted even at 72 hours after IA training (Fig 2B). All of the genes that were up-regulated 24 hours post IA training, were still up-regulated even at 72 hours. In addition to the increased expression of *MCT1* (33 ± 11.2%), *MCT4* (38 ± 13.1%), *Glut1* (24 ±5.1%), *Glut3* (25 ± 3.8%) and *Na/K alpha2* (22 ± 6.7%), a significant induction of lactate dehydrogenase A *Ldha* (22 ± 5.2%) and *Ldhb* (34 ± 9.3%) was observed at this time point (n = 7).

**Fig 3:**
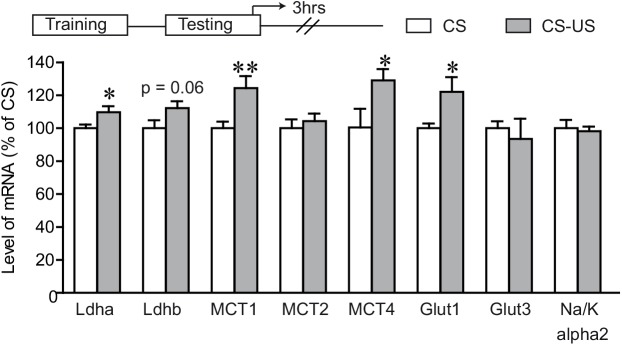
Expression of ANLS related genes following IA long term memory. Gene expression profile for ANLS related genes was assessed in the dorsal hippocampus 3 hours following retention testing using quantitative Q-PCR. Results are expressed as percentage of control values (CS group) and are means ± SEM. Data were statistically analyzed using two-tailed Student’s t test, * P < 0.05, ** P < 0.01 vs CS group, n = 10/ group.

The hippocampus plays a critical role not only in memory consolidation but also in memory retrieval [42,43]. Moreover, a recent study has shown that memory consolidation may have different metabolic demands compared to memory retrieval [44]. Therefore, in order to understand the contribution of neuron-glia metabolic coupling in memory retrieval, we further extended our gene expression analysis to IA post retention testing time point. We found that some of the ANLS related genes that were induced 24 hours after training (Fig 1A) continue to be highly expressed even at 3 hours post retention testing (Fig 3). Interestingly, in addition to the significant 10 ± 3.6% induction of *Ldha*, we found that genes expressed by astrocytes, namely *MCT1* (24 ± 7.2%), *MCT4* (29 ± 6.8%) and *Glut1* (22 ± 8.8%) were significantly induced in the CS-US group.

**Fig 4:**
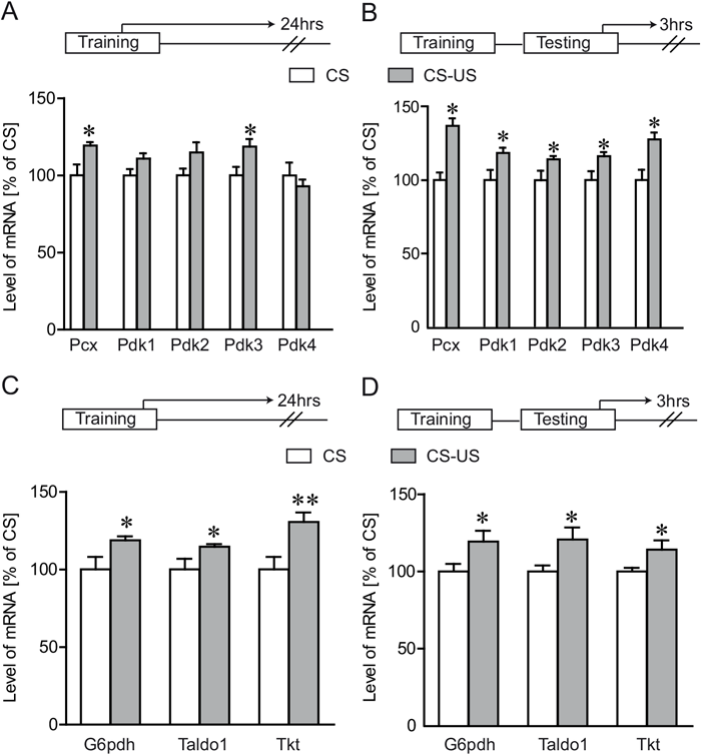
Differential expression of pentose phosphate pathway and pyruvate metabolism following IA learning. Dorsal hippocampal mRNA levels for pyruvate metabolism (**A** and **B**) and pentose phosphate pathway (**C** and **D**) related genes were assessed by Q-PCR following IA task. Gene expression profile was assessed in animals sacrificed 24 hours following IA training (A and C) or 3 hours following IA testing (B and D). A 19 ± 2.2% increase of pyruvate carboxylase (*Pcx*) was seen in the CS-US group 24 hours post training. Furthermore, this increase was seen even 3 hours post-retention testing (37 ± 5.1%). The expression of pyruvate dehydrogenase kinases (*Pdk*’s) was modulated following IA learning. A 19 ± 4.9% increase in dorsal hippocampal expression of pyruvate dehydrogenase kinase 3 (*Pdk3)* and an almost significant induction of pyruvate dehydrogenase kinase 1 and 2 (*Pdk1and Pdk2*) was seen in the CS-US group, 24 hours post training (For *Pdk1*, P = 0.066; For *Pdk2*, P = 0.085). When assessed 3 hours post retention testing, we found significant induction of all the *Pdk’*s in the CS-US group (a 19 ± 3.5% increase of *Pdk1*; a 14 ± 2.1% for *Pdk2;* a 16 ± 2.7% for *Pdk3;* a 28 ± 4.6% for *Pdk4*). In contrast to the above data, when tested at 72 hours post training, no significant gene expression differences were seen across the two groups (p<0.05, data not shown). A significant up-regulation of glucose-6-phosphate dehydrogenase *(G6pdh)*, transaldolase 1 (*Taldo1*) and transketolase (*Tkt*) was seen in the dorsal hippocampus 24 hours post training (a 19 ± 2.5% increase of *G6pdh*, a 15 ± 1.7% increase of *Taldo*, a 31 ± 6% increase of *Tkt*) as well as 3 hours post retention testing (a 19 ± 7% increase of *G6pdh*, a 21 ± 7.8% increase of *Taldo1*, a 14 ± 5.9% increase of *Tkt*). Results are expressed as percentage of control values (CS group) and are the means of ± SEM. Data were statistically analyzed using two-tailed Student’s t test, * P < 0.05, ** P < 0.01, vs CS group, n = 8/group.

We further extended our analysis to examine pyruvate metabolism and pentose phosphate pathway following IA learning (Fig 4). We observed an increased expression of genes related to pyruvate metabolism following the IA task (Fig 4A and 4B) as well as to the pentose phosphate pathway (Fig 4C and 4D), at both 24 hours post-training and 3 hours post-retention testing times. These genes include the astrocytic pyruvate carboxylase (*Pcx*), pyruvate dehydrogenase kinases 1–4 (*Pdk1-4*), glucose 6-phosphate dehydrogenase (*G6pdh*), transaldolase 1 (*Taldo1*) and trasketolase (*Tkt*).

As a whole, the gene expression analyses presented here demonstrate learning-induced increased dorsal hippocampal expression of genes involved in neuron-glia metabolic cooperation after IA learning, revealing the contribution of these genes to the formation of IA memory. The work reported in this article does not have the ambition to provide support for the existence of the ANLS; it simply describes the fact that some genes whose expression is modulated by learning are related to the ANLS model, but that others, also regulated by learning are not.

### Glycogen metabolism-related gene expression following IA learning

**Fig 5:**
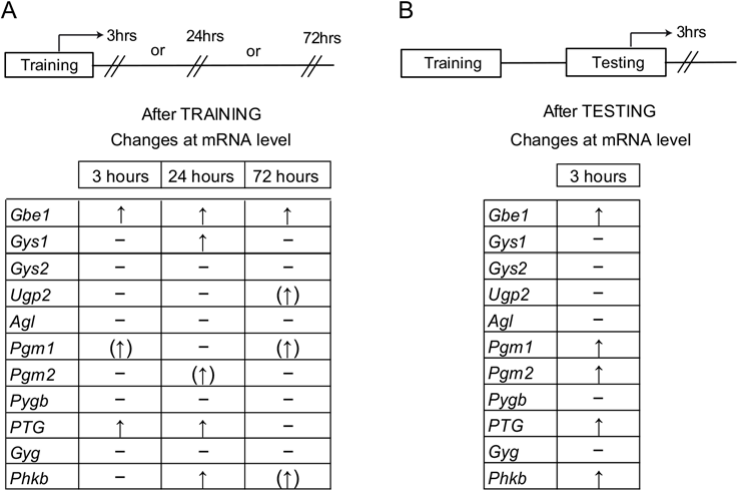
Glycogen regulation following IA learning. The table summarizes the changes seen in the mRNA expression of genes related to glycogen metabolism following IA task at 3, 24 or 72 hours after training **(A)** or 3 hours after testing **(B)**. An upright arrow indicates significant induction of the gene in dorsal hippocampus of the CS-US group (P < 0.05, versus CS group, n = 7-8/group) whereas an upright arrow in brackets indicates an almost significant induction (i.e. P values very close to significance [P<0.05]) and a “-“entry indicates no significant difference across the two groups. Data were statistically analyzed using two-tailed Student’s t test. For abbreviations, see materials and methods section.

Recently, we have shown that astrocytic glycogen metabolism in the hippocampus plays a critical role in LTM formation [13]. We therefore performed a detailed gene expression analysis of genes related to glycogen metabolism following an IA task. Several genes related to both glycogen synthesis and degradation were modulated following IA learning. Fig 5 summarizes the modulation of glycogen metabolism related genes in the dorsal hippocampus across the different experimental conditions used in the study i.e. 3, 24 and 72 hours post-training (Fig 5A) and 3 hours after post-retention testing (Fig 5B). The dorsal hippocampus of the CS-US group demonstrated a 21 ± 8.3% increase of protein targeting to glycogen (*PTG*) mRNA as early as 3 hours post training. This induction persisted 24 hours post-training (18 ± 7.6%) as well as 3 hours post retention testing (9 ± 2.1%). However, this differential expression of *PTG* was lost 72 hours post-training (P> 0.05). We also observed a 16 ± 3.6% increase in hippocampal expression of glycogen branching enzyme 1 (*Gbe1*) in the CS-US group 3 hours post training, and this induction persisted both 24 hours (23 ± 4.5%) as well as 72 hours post-training (26 ± 3.3%). Furthermore, this up-regulation of *Gbe1* was also seen at 3 hours post-retention testing (14 ± 4.3%). In addition, expression of phosphorylase b kinase (*Phkb*), which has a major regulatory role in the breakdown of glycogen was increased by 15 ± 5.7%, 24 hours post training and by 20 ± 5.5% 3 hours post-retention testing. Increased expression (31 ± 6%) of the muscle isoform of glycogen synthase (*Gys1*) that catalyzes the progressive extension of glycogen chain by adding successive glucose molecules was seen only at 24 hours post IA training but not at other time points (Fig 5). The expression of mRNA for other genes such as the brain isoform of glycogen phosphorylase (*Pygb*), responsible for glycogen degradation, did not differ across the two groups for all the tested time points (Fig 5).

### Induction of proteins expressed by astrocytes following IA learning

**Fig 6:**
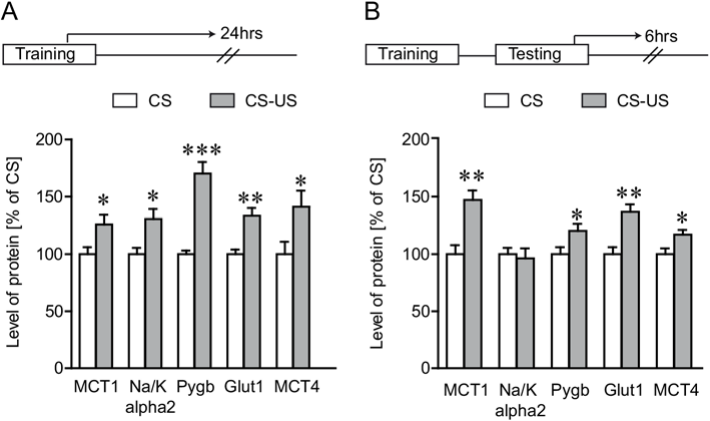
Analysis of selected proteins following IA learning. Dorsal hippocampal protein expression levels were measured using extracts from animals sacrificed 24 hours following IA training **(A)** or 6 hours following IA retention testing **(B)**. Protein expression level of MCT1, Na/K alpha2, Glut1, MCT4 and Pygb were assessed by the western blotting. Results are expressed as percentage of control value and are means ± SEM of OD units from each group normalized to β-actin, except for Glut1 which was normalized to β-tubulin. Asterisks indicate a significant difference (Student’s t-test; * P < 0.05, ** P < 0.01, *** P < 0.001 vs CS group, n = 7 per group).

To determine whether the protein levels for a selection of genes related to energy metabolism correspondingly changed with mRNA induction, we performed quantitative western blot analysis on dorsal hippocampal protein extracts after the behavioral test (Fig 6). Groups of mice received IA training and protein concentrations were measured 24 hours after training and 6 after retention testing and compared with controls (CS group). As shown in Fig 6A, dorsal hippocampus of CS-US animals show an increased expression of MCT1 (26%), MCT4 (41%), Glut1 (33%), Na/K alpha2 (30%) and a substantial 70% increased expression of Pygb 24 hours post IA training. Furthermore, this increased protein expression was persistent even at 6 hours post retention testing [(Fig 6B); for MCT1 (47%), MCT4 (17%), Glut1 (36%) and Pygb (20%)].

### Memory impairment in MCT1 heterozygous mice

Recently it was shown that interference of lactate transfer from astrocytes to neurons via the MCTs (MCT1, 2 and 4) impairs long term memory [13]. Complementing the above findings, in our study we found that MCT1, an ANLS related gene was highly induced in the dorsal hippocampus following IA learning (Figs 2 and 6). In order to investigate the behavioral consequences of down regulating *MCT1*, we used mice heterozygous for a *MCT1* targeted disruption (MCT1^+/−^) to examine the implication of this gene in the IA cognitive task. The MCT1^+/−^ mice have a 50% reduction of the MCT1 protein in total brain extracts [17] and a 40% decrease in the hippocampus (data no shown). While MCT1^-/-^ mice die at an early embryonic stage, MCT1^+/-^ mice, which have been previously characterized, show no apparent abnormalities at the morphological and functional levels under standard conditions [17]. Before testing the MCT1^+/-^ in cognitive behavior, we also examined their motor and sensory functions and found no difference compared to their wild-type littermates (data not shown).

**Fig 7:**
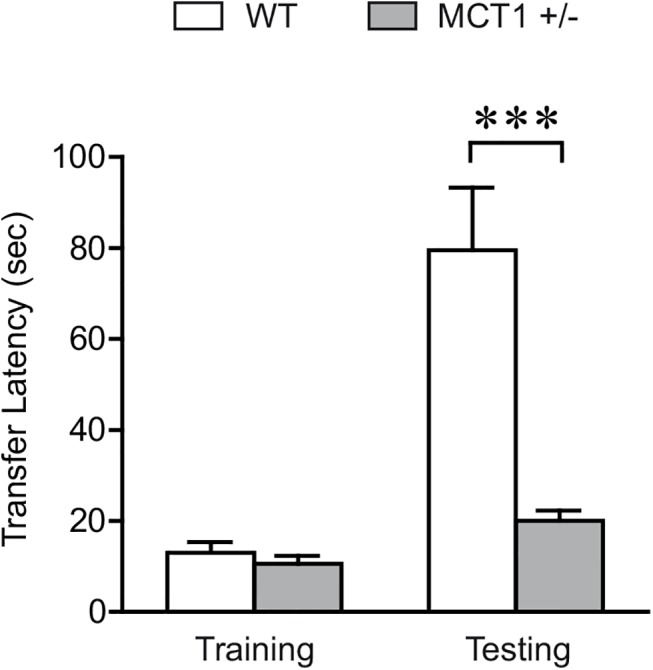
Down regulation of MCT1 impairs long term memory in MCT1 ^+/-^ heterozygous mice. Step-through latencies were measured during IA training and retention testing (24 hours after the foot shock). Results are shown as mean ± SEM transfer latency responses. Baseline latency did not differ significantly across the two groups (p>0.05). MCT1^+/-^ mice depicted a significantly reduced transfer latency time for the retention trial than the wild-type (WT) littermates (Two-way ANOVA followed by Bonferroni post hoc test; ***P < 0.001, MCT1^+/-^: n = 14; WT littermates: n = 14).

When tested for context-dependent avoidance behavior, MCT1^+/-^ mice exhibited 4 times lower transfer latencies (19.9 ± 2.3 vs 79.3± 14.3 sec) for retention trial than the controls indicating impairment of long term memory (Fig 7). Two-way ANOVA followed by Bonferroni post hoc test revealed that MCT1^+/-^ mice have significantly lower transfer latency on the retention testing day (P < 0.001) whereas baseline latency to enter the dark chamber did not differ between the two groups (p > 0.05). The above results point to a critical role of MCT1 for long term memory formation.

## Discussion

The data reported here show that genes encoding proteins involved in brain energy metabolism, including sets of genes implicated in neuron-glia metabolic coupling and in several cases specific to astrocytic metabolic pathways are regulated during learning. These changes were seen in dorsal hippocampus, a region that we found metabolically active during the retrieval of IA learning task.

Using [^14^C] 2DG as an indicator of brain activity, we mapped glucose utilization in brain areas following IA learning and found that hippocampus, BLA, ACC and IL and PL cortex were metabolically activated upon IA memory retrieval. Consistent with previous studies showing an active role of the above mentioned regions in IA learning [29,32,40,45,46], our results show that retrieval of IA memory places a higher metabolic demand (higher glucose utilization) on the very same regions. The [^14^C] 2DG technique gives an excellent mapping of the regional metabolic activity, but does not distinguish between different compartments such as neuronal and glial cells. With respect to this, studies have shown that at rest, astrocytes of the rat brain are responsible for approximately half of glucose utilization [47,48], and that this proportion increases even further upon functional brain activation [47]. These observations support the view that functional imaging experiments based on glucose analogue extraction may predominantly reflect the metabolic activity of the astrocytic network [5,49]. In light of the hypothesis that glucose positron emission tomography (PET) signals mainly reflect glucose consumption in astrocytes rather than in neurons, an interesting observation of our study is the learning-specific induction of the genes expressed by astrocytes (*MCT1*, *MCT4*, *Glut1*, *Na/K alpha2* and *Pcx*) in the metabolically active dorsal hippocampus. A recent article by Lundgaard et al. [50], using a novel near infrared 2-deoxyglucose optical imaging dye, IRDye 800CW 2-DG, has suggested a predominant uptake of glucose into neurons rather than astrocytes, not in support of the ANLS. The study by Lundgaard et al. has used a dye, IRDye 800CW 2-DG which, as stated by their developers, Kovar et al. (2009), detects uptakes of Glut1/IRDye 800CW 2-DG complexes (most likely through an endocytotic process) into cells rather than glucose transport [51]. This is not surprising as the molecular weight of the dye is 1166 compared to 180 for glucose.

It is therefore likely that what the article by Lundgaard et al. reports is an endocytotic process of a glucose transporter, possibly an interesting observation, however unrelated to the process of glucose transport into neural cells.

Pertaining to the nature of the cell type predominantly taking up circulating glucose into the brain parenchyma Cholet et al. and Voutsinos-Porche et al. have shown that astrocytic glutamate transporters represent the key signal for glucose uptake into these cells as predicted by the ANLS [52,53]. Also further stressing the importance of glucose uptake into astrocyes compared to neurons, transgenic mice with an haploinsufficiency of glucose transporters Glut3 (the neuron-specific glucose transporter) do not present a pathological phenotype and have a normal cerebral glucose utilization [54,55], while haploinsufficiency of Glut1, which is absent in neurons and expressed in astrocytes, produces a severe neurological phenotype [56].

The induction of genes related to brain energy metabolism in the hippocampus after IA training is selective for the shock–context association and is not an effect of novelty alone nor shock alone, as shown here for ANLS-related genes. These findings support previous studies showing that the hippocampal response is selective for learning [31,57]. We observed no changes in the expression level of the same set of genes in somatosensory cortex, further highlighting the regional specificity of learning associated changes in the CS-US group. Together, these results indicate that fear-based contextual learning involves selective activation of genes related to brain energy metabolism within restricted brain regions.

Our results also show that following IA learning there is marked hippocampal induction of genes related to the ANLS (Fig 2). Despite the fact that considerable experimental evidence accrued over twenty years supports the existence of the ANLS, some discordant views on certain of its aspects are still occasionally presented on theoretical or modeling grounds [58–61]. These views have been regularly addressed and discussed (see e.g. Pellerin and Magistretti 2012 [7]) most recently in Magistretti and Allaman 2015 [6].

Regarding genes involved in the ANLS we describe here a significant induction of the monocarboxylate transporters *MCT1* and *MCT4*, which are expressed in astrocytes, following IA training as well as IA testing, whereas the neuronal transporter *MCT2* remained unchanged. The absence of changes in the expression of mRNA encoding for MCT2 may appear surprising as far as the ANLS model is concerned. However it is conceivable that the actual uptake capacity prior to learning is not maximal and that the expression level of MCT2 in neurons is sufficient to accommodate an increased release by astrocytes. Of note are the observations by Pellerin and collaborators that MCT2 expression can be controlled through translational mechanisms [62,63] and that activation promotes the translocation at the postsynaptic membrane of MCT2 from an intracellular cytoplasmic pool [64]. The observation of an increased *GLUT3* expression is of interest, as it indicates that, even though glucose uptake in mainly determined by hexokinase rather than by transporters, possibly there is indeed an increased glucose use by neurons following learning. Since an important proportion of glucose is used by neurons in the pentose phosphate shunt to produce reducing equivalents [65,66], it may be that the increased reducing power afforded by an upregulation of neuronal glucose metabolism matches an increased necessity of reactive oxygen species scavenging.

We also found that mice heterozygous for *MCT1* have severe LTM deficits when tested in the same behavioral paradigm (Fig 7). These mice show only minor differences in blood metabolic parameters, compared to wild-type littermates [17], making it unlikely that systemic changes affected memory performance. These data are consistent with previous reports showing that lactate transport through astrocytic MCT1 and MCT4 is critical for long-term memory formation [13] and further provide not only correlative but also functional evidence for a role of MCT1 in hippocampal learning.

Another gene that we found to be induced following IA learning is the astrocyte-enriched Na/K alpha2 indicating its importance in learning and memory. Interestingly, reduction in the expression of *Na/K alpha2* was reported to result in hippocampal learning deficits [67,68]. Furthermore, astrocytic glucose transporter *Glut1* was induced both following IA training and testing in contrast to neuronal glucose transporter *Glut3* which was induced only following IA training but not post IA testing. Overall, these observations support the notion of an important role of astrocyte-neuron lactate shuttling in neuronal functions and point towards its critical role in IA LTM formation.

An important source of lactate in the brain is glycogen. Astrocytic glycogen can be mobilized via glycogenolysis [10,69]. The importance of glycogenolysis as an energy source must be short term, since glycogen stores in the brain are depleted rapidly (within minutes) if glycogen serves as the only metabolic substrate. Supplementing to this fast process, it is interesting to note that among all the pathways we analyzed, genes related to glycogen metabolism, in particular synthesis, were the only ones that were up regulated as early as 3 hours post-IA training. Consistent with the previous reports that *PTG* induction results in increased glycogen synthesis and storage in cells and tissues with high levels of PTG [70,71], we also observed induction of *PTG* as early as 3 hours post-IA training. Complementing the recent finding that mice lacking glycogen synthase in brain exhibit deficits in cognitive learning [72], in our study we found a learning specific induction of *Gys1* only in the CS-US group 24 hours following IA training. Furthermore, we found a persistent up-regulation (from 3 to 72 hours post-IA training) of *Gbe1*, a gene that is required for sufficient glycogen accumulation. *Pygb* was induced following IA training and testing at the protein level but not at the mRNA level suggesting posttranslational modifications. Overall, complementing the importance of glycogen mobilization in memory formation shown in previous reports [14,73] our results are consistent with the existence of an increased glycogen metabolism, and further support the role of glycogen in learning and memory.

The temporal profiles of gene induction after IA training exhibit two important features: first, the onset of enhanced gene expression of the metabolic pathways occurs in a delayed manner (24 hours) and second, the changes are sustained for several hours (at least 72 hours) following IA training. Together with studies reported earlier [74–76], our findings are consistent with the hypothesis that LTM storage is achieved by recurrent rounds of consolidation-like mRNA and protein synthesis-dependent processes [77,78].

The gene expression data presented in this study point towards the key metabolic event, lactate production following IA learning and support the hypothesis that following neuronal activity, lactate produced via glycolysis and glycogenolysis in astrocytes not only provides energy to activated neurons, but is also needed for the establishment of LTM. This could be mediated by a recently discovered molecular mechanism that involves NMDA receptor modulation [79].

Both glycogen metabolism and glycolysis by ANLS result in lactate production thereby positioning the monocarboxylate transporters as an essential bridge for astrocyte-neuron interaction following learning. This key role of MCTs was highlighted in our findings where we observed that LTM formation is associated with induction of MCT1 in the hippocampus following learning. Further complementing this finding, by using genetic manipulation as a tool, we observed that a partial knockdown of MCT1 results in impaired IA memory in mice. These findings provide an insight into the behavioral/cognitive consequences of inactivating the expression of a glia-specific gene and contribute to our understanding of the relationship between brain energy metabolism and cognition. It should however be acknowledged that for most studied enzymes increases in both mRNA and protein (determined for MCT1, MCT4, Na/K alpha2, Glut1 and Pygb) although statistically significant were small in percentage, potentially raising the question of the biological/metabolic meaning of such changes. In this context, one should mention that an increase as modest as 20% of MCT1 in the hippocampus was shown to be crucial for the establishment of LTM in IA in rat [13], suggesting that expression changes reported here are biologically relevant.

An increased expression of enzymes related to pyruvate metabolism as well as the pentose phosphate pathway was also seen following IA learning, suggesting that learning overall modulates several energy metabolism related pathways. However, the precise role for such alterations remains to be established.

As a whole, the results obtained in this study demonstrate modulation of hippocampal gene expression following learning and provide evidence for the existence of adaptive mechanisms related to neuron-glia metabolic coupling associated with memory formation. The identification of metabolic genes induced following learning, provides novel insights into molecular aspects of memory and highlights the indispensable role of astrocytes and neuronal–astrocyte metabolic coupling in learning and memory consolidation. These results may have important implications for conditions involving deficits in cognitive performance such as Alzheimer’s disease, aging, and dementia [80–82].

## Supporting Information

S1 Table Gene expression analysis in the somatosensory cortex. Somatosensory tissues in CS and CS-US animals were collected 24 hours following inhibitory avoidance and mRNA expression levels for ANLS related genes were assessed by quantitative Q-PCR. Results are expressed as percentage of control values (CS group) and are means ± SEM (n = 7–8 per group). Data were statistically analyzed using two-tailed Student’s t test and no statistical differences were observed between CS and CS-US groups. The statistical details are provided in the table.
(PDF)

S2 Table Shock alone does not induce the expression of genes related to ANLS. Dorsal hippocampal tissues in CS and US (Shock only) animals were collected 24 hours following inhibitory avoidance and mRNA expression levels for ANLS related genes were assessed by quantitative Q-PCR. Results are expressed as percentage of control values (CS group) and are means ± SEM (n = 8 per group). Data were statistically analyzed using two-tailed Student’s t test and no statistical differences were observed between CS and US (Shock only) groups. The statistical details are provided in the table.
(PDF)
