# Supplementary material for: Learning-Induced Gene Expression in the Hippocampus Reveals a Role of Neuron -Astrocyte Metabolic Coupling in Long Term Memory
Source: PLoS One. 2015 Oct 29;10(10):e0141568. doi: 10.1371/journal.pone.0141568 (PMC4625956; doi:10.1371/journal.pone.0141568)
Supplement: S1 Table — Somatosensory tissues in CS and CS-US animals were collected 24 hours following inhibitory avoidance and mRNA expression levels for ANLS related genes were assessed by quantitative Q-PCR. Results are expressed as percentage of control values (CS group) and are means ± SEM (n = 7–8 per group). Data were statistically analyzed using two-tailed Student’s t test and no statistical differences were observed between CS and CS-US groups. The statistical details are provided in the table. (PDF) [file pone.0141568.s001.pdf]

S1 Table:

Gene expression analysis in the somatosensory cortex

	CS	CS-US	
	Mean ± SEM, N	***Mean ± SEM, N***	***t ,df***
**Astrocyte Neuron Lactate Shuttle**			
Ldha	1.000 ± 0.088 N=7	1.067 ± 0.072 N=8	t=0.593, df=13
Ldhb	1.000 ± 0.095 N=7	1.184 ± 0.084 N=8	t=1.455, df=13
MCT1	1.000 ± 0.044 N=7	0.986 ± 0.030 N=8	t=0.276, df=13
MCT2	1.000 ± 0.056 N=7	0.922 ± 0.031 N=8	t=1.258, df=13
MCT4	1.000 ± 0.080 N=7	0.990 ± 0.059 N=8	t=0.100, df=13
Glut1	1.000 ± 0.062 N=7	0.980 ± 0.031 N=8	t=0.306, df=13
Glut3	1.000 ± 0.073 N=7	1.066 ± 0.090 N=8	t=0.556, df=13
Na/K alpha2	1.000 ± 0.039 N=7	0.898 ± 0.082 N=8	t=1.074, df=13
