# Supplementary material for: Learning-Induced Gene Expression in the Hippocampus Reveals a Role of Neuron -Astrocyte Metabolic Coupling in Long Term Memory
Source: PLoS One. 2015 Oct 29;10(10):e0141568. doi: 10.1371/journal.pone.0141568 (PMC4625956; doi:10.1371/journal.pone.0141568)
Supplement: S2 Table — Dorsal hippocampal tissues in CS and US (Shock only) animals were collected 24 hours following inhibitory avoidance and mRNA expression levels for ANLS related genes were assessed by quantitative Q-PCR. Results are expressed as percentage of control values (CS group) and are means ± SEM (n = 8 per group). Data were statistically analyzed using two-tailed Student’s t test and no statistical differences were observed between CS and US (Shock only) groups. The statistical details are provided in the table. (PDF) [file pone.0141568.s002.pdf]

S2 Table:

Shock alone does not induce the expression of genes related to ANLS

	US (Shock only)	**CS**	
	Mean ± SEM, N	***Mean ± SEM, N***	***t ,df***
Astrocyte Neuron Lactate Shuttle			
Ldha	*0.985 ± 0.030 N=8*	1.000 ± 0.025 N=8	t=0.383, df=14
Ldhb	*1.153 ± 0.054 N=8*	1.000 ± 0.047 N=8	t=2.135, df=14
MCT1	*1.002 ± 0.026 N=8*	1.000 ± 0.040 N=8	t=0.034, df=14
MCT2	*1.043 ± 0.025 N=8*	1.000 ± 0.026 N=8	t=1.182, df=14
MCT4	*0.965 ± 0.058 N=8*	1.000 ± 0.132 N=8	t=0.241, df=14
Glut1	*1.084 ± 0.045 N=8*	1.000 ± 0.042 N=8	t=1.362, df=14
Glut3	*1.080 ± 0.088 N=8*	1.000 ± 0.069 N=8	t=0.716, df=14
Na/K alpha2	*1.127 ± 0.098 N=8*	1.000 ± 0.037 N=8	t=1.209, df=14
